# Extraskeletal myxoid chondrosarcoma of the penis: Description of a rare tumor arising in an unusual location

**DOI:** 10.1016/j.eucr.2022.102151

**Published:** 2022-07-04

**Authors:** Khaled A. Murshed, Bashar Aldaraiseh, Aalaa Kambal, Alaaldin Badawi, Issam Albozom

**Affiliations:** a Department of Anatomic Pathology, Hamad Medical Corporation, Doha, Qatar; b Department of Diagnostic Radiology, Hamad Medical Corporation, Doha, Qatar; c Department of Urology, Hamad Medical Corporation, Doha, Qatar

**Keywords:** Extraskeletal myxoid chondrosarcoma, Penis, Perineum, Lung, Metastasis

## Abstract

Extraskeletal myxoid chondrosarcoma (EMC) is a rare malignant soft tissue tumor that most commonly arises in the extremities. Its occurrence in the genitourinary tract is extremely uncommon. We present an 82-year-old man who was found to have incidental pulmonary metastasis by imaging studies. The biopsy findings from the lung were in favor of metastatic EMC. The primary mass was found to be located at the penile root, which was confirmed to be EMC. This case adds to the few reported cases of EMC arising in the perineal region, and sheds light on the clinical behavior of this soft tissue tumor.

## 1 Introduction

EMC is a rare malignant soft tissue tumor that accounts for <1% of all soft tissue sarcomas.^1^^,^^2^ It has a predilection for the deep soft tissue of the extremities, particularly the thighs.^1^^,^^2^ Its occurrence in the perineal region is extremely rare with only handful cases reported.^3^ EMC is a low-grade sarcoma that has high risk for local recurrence and distant metastasis, most commonly to the lungs.^2^^,^^4^ Even with the presence of metastatic disease, EMC has good overall survival rates.^4^ Herein, we present an 82-year-old gentleman who had widespread lung metastasis as the initial manifestation of EMC. The primary tumor was found to be located at the penis with extension into the perineum and pubic bone.

## 2 Case presentation

**Fig. 1:**
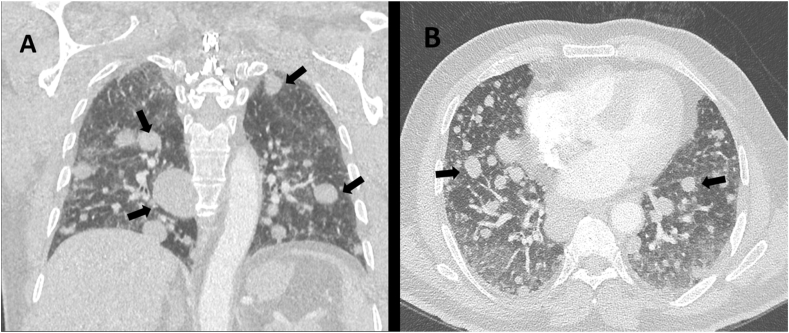
Computed Tomography scan of the thorax. **A**, coronal and **B**, axial planes showing multiple variable-sized solid pulmonary nodules (black arrows) at both lung parenchyma, in keeping with metastasis.

**Fig. 2:**
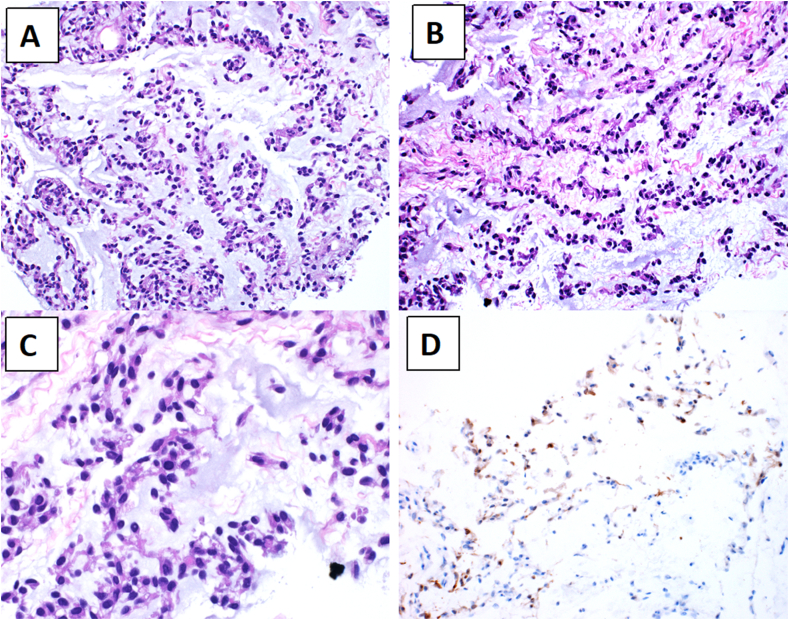
Microscopic and immunohistochemical features of the tumor. **A** and **B**, the tumor cells are arranged in cords and trabeculae that are embedded in myxoid matrix (Hematoxylin & eosin stain x200). **C**, High power view shows uniform oval to spindle tumor cells that have uniform chromatin and eosinophilic cytoplasm (Hematoxylin & eosin stain x400). **D**, the tumor cells show focal nuclear and cytoplasmic staining for S100 protein (Immunohistochemistry x200).

An 82-year-old gentleman presented with an episode of acute pancreatitis for two days duration. CT-scan of abdomen and chest revealed an incidental finding of multiple bilateral ill-defined reticulonodular lung infiltrates, in keeping with metastasis (Fig. 1A and B). A CT-guided core biopsy of the lung lesions showed a myxoid neoplasm composed of bland spindle to oval shaped cells that have hyperchromatic nuclei and pale to eosinophilic cytoplasm. The cells were arranged in cords and reticular arrays (Fig. 2A–C). By immunoperoxidase stains, the tumor cells were focally reactive for S100, but negative for CKAE1/AE3, EMA, TTF1, Napsin-A, SMA, Desmin, CD34, SATB2, MDM2 and CDK4 (Fig. 2D). Based on these findings, the diagnosis of myxoid sarcoma with features favoring metastatic EMC was rendered. Fluorescence in situ hybridization (FISH) analysis was performed on formalin-fixed paraffin-embedded tissue (FFPE). The testing was performed using *EWSR1* Break Apart Probe (Abbott laboratories, Illinois, U.S.A). *EWSR1* gene rearrangement was detected. Next generation sequencing (NGS) failed to detect any specific gene fusions.

**Fig. 3:**
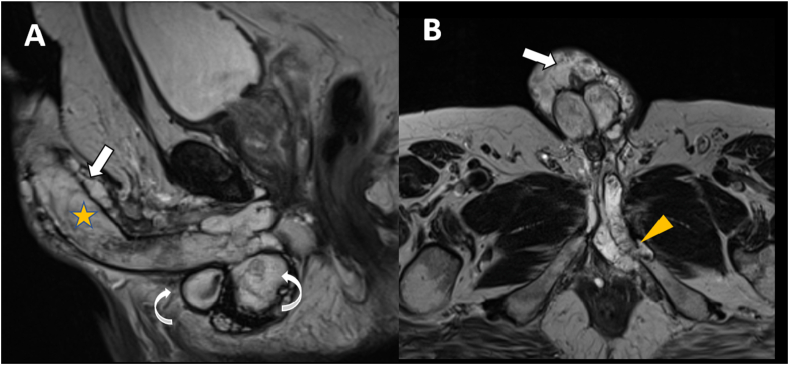
Magnetic Resonance Imaging of the pelvis. **A**, Sagittal T2 weighted and **B**, axial T2 weighted MR images showing diffuse infiltrative T2 bright signal intensity lesion along the whole penile shaft (yellow star) extending into superficial extra-albugineal tissue (white bold arrows) and involving the left inferior pubic ramus (yellow arrowhead). In addition, multiple lobulated T2 bright signal intensity lesions inferior to the penile root (curved white arrows). (For interpretation of the references to colour in this figure legend, the reader is referred to the Web version of this article.)

A thorough physical examination of the trunk and extremities revealed a subcutaneous firm immobile mass at the base of penile shaft extending to the scrotum. MRI of the penis showed an infiltrative soft tissue multinodular lesion within the base of the penile shaft involving the corpus spongiosum and left corpus cavernosum, measuring 8 cm in maximum dimension (Fig. 3A and B). The lesion extended to the left side of perineum and involved the left pubic bone body and ramus. A core biopsy was taken from the penile lesion, which showed a tumor with similar morphological and immunohistochemical features to the pulmonary lesions. The diagnosis of EMC of the penis was confirmed.

The patient was started on Pazopanib 800 mg daily, which was complicated by fatigue, high blood pressure, endocrine failure, and diarrhea. Follow-up PET-scan showed stable lung lesions and minimal partial metabolic response at the root of the penis. Pazopanib was halted for 2 months, and then continued. The patient is now on regular follow-up at 3-months-interval with no signs of progression.

## 3 Discussion

Initially described as chordoid sarcoma by Enzinger and Shiraki in 1972,^1^ extraskeletal myxoid chondrosarcoma (EMC) is a rare soft tissue tumor that arises most commonly in the deep soft tissue of extremities.^1^^,^^2^ Its occurrence in the genitourinary tract is very unusual with only few cases reported.^3^ In our case, the tumor was located at the base of the penile shaft and extended to the perineum and pubic bone.

EMC is currently classified by the World Health Organization (WHO) classification under the category of tumors of uncertain differentiation.^2^ Despite its name, well-formed hyaline cartilage is never seen in this tumor. Microscopically, EMC is a tumor characterized by having multinodular and lobular growth pattern. The tumor cells are interconnected to form cords and reticular networks in pale to blue myxoid matrix. They have uniform oval to spindle shaped nuclei with modest amount of eosinophilic cytoplasm. In our case, the tumor presented initially with widespread lung metastasis, which posed a diagnostic challenge. A core biopsy was taken from the lung lesions, which showed a myxoid tumor composed of mildly atypical oval to stellate shaped cells arranged in cords and reticular arrays.

The differential diagnosis based on the site and morphology included primary pulmonary mucinous adenocarcinoma, metastatic mucinous carcinoma and primary pulmonary myxoid sarcoma (PPMS). Epithelial markers including CKAE1/AE3 and EMA were negative, which ruled out the possibility of primary/metastatic mucinous carcinoma. In addition, lung adenocarcinoma markers (TTF-1 and Napsin-A) were also negative. PPMS is a rare primary low-grade myxoid sarcoma of the lung that very much resembles EMC at the morphologic and genetic levels. Like EMC, PPMS is composed of lobules of tumor cells arranged in cords and lace-like strands of uniform cells in a myxoid matrix. It has also frequent *EWSR1* gene rearrangement. However, PPMS has a characteristic stroma rich in lymphoplasmacytic infiltrate, which was lacking in our case. Moreover, PPMS is frequently reactive for EMA, which was negative in our case.

EMC harbors a characteristic and diagnostically helpful *NR4A3* gene rearrangements.^2^^,^^5^ They are present in >90% of EMCs. *NR4A3* gene, which is located at chromosome 9q22, fuses with different partners. The most frequent is *EWSR1* at 22q12.2 followed by *TAF15* at 17q12.^2^^,^^5^ In our case, *EWSR1* gene rearrangement was detected by FISH break apart probe, however, NGS failed to detect any specific gene fusions. In addition to the diagnostic utility of these genetic rearrangements, they have a prognostic value. It has been found that EMCs with variant non-*EWSR1* gene fusions tend to show more aggressive outcomes than *EWSR1-NR4A3* rearranged tumors.^2^

Generally, EMC has good prognosis with 5-year overall survival rate of >80%.^2^^,^^4^ Despite the high risk of local and distant recurrence, patients have prolonged survival even with the presence of metastatic disease.^4^ In our case, the initial manifestation of the disease was the widespread bilateral lung metastasis, that was discovered incidentally by imaging. This was an unusual event that posed a diagnostic challenge.

## 4 Conclusion

In summary, we are presenting a case of EMC of the penis that was initially presented with lung metastasis. EMCs of the penis and perineum are very rare. Proper identification is essential as EMC carries good clinical outcome even in the face of metastatic disease.

## Funding

Open Access funding provided by the 10.13039/100019779Qatar National Library.

## Author contributions

KM wrote the first draft of the manuscript and performed literature review. BA assisted in manuscript writing and literature review. AK provided radiology images and reviewed the manuscript. AB provided clinical information and reviewed the manuscript. IB supervised the study and reviewed the manuscript.

## Ethics approval

The Institutional Review Board at Hamad Medical Corporation approved publication of this article under the number (MRC-04–22-238).

## Consent for publication

Informed consent from the participant has been waived by Institutional Review Board.

## Declaration of competing interest

The authors declare that they have no known competing financial interests or personal relationships that could have appeared to influence the work reported in this paper.
